# Endogenous Oxytocin Release Eliminates In-Group Bias in Monetary Transfers With Perspective-Taking

**DOI:** 10.3389/fnbeh.2018.00035

**Published:** 2018-03-05

**Authors:** Elizabeth T. Terris, Laura E. Beavin, Jorge A. Barraza, Jeff Schloss, Paul J. Zak

**Affiliations:** ^1^Center for Neuroeconomics Studies, Claremont Graduate University, Claremont, CA, United States; ^2^Department of Biology, Westmont College, Santa Barbara, CA, United States

**Keywords:** prosociality, neuroendocrinology, selfishness, monetary exchange, bias

## Abstract

Oxytocin (OT) has been shown to facilitate trust, empathy and other prosocial behaviors. At the same time, there is evidence that exogenous OT infusion may not result in prosocial behaviors in all contexts, increasing in-group biases in a number of studies. The current investigation seeks to resolve this inconsistency by examining if endogenous OT release is associated with in-group bias. We studied a large group of participants (*N* = 399) in existing groups and randomly formed groups. Participants provided two blood samples to measure the change in OT after a group salience task and then made computer-mediated monetary transfer decisions to in-group and out-group members. Our results show that participants with an increase in endogenous OT showed no bias in monetary offers in the ultimatum game (UG) to out-group members compared to in-groups. There was also no bias in accepting UG offers, though in-group bias persisted for a unilateral monetary transfer. Our analysis shows that the strength of identification with one’s group diminished the effects that an increase in OT had on reducing bias, but bias only recurred when group identification reached 87% of its maximum value. Our results indicate that the endogenous OT system appears to reduce in-group bias in some contexts, particularly those that require perspective-taking.

## Introduction

As with all social animals, it is the nature of humans to form groups. People more readily affiliate with those who share common traits or behaviors (Prentice et al., 1994). Group bonding can benefit members in a group by promoting cooperation and altruism (Penner et al., 2005; Hein et al., 2010; Weller and Hansen Lagattuta, 2013), but it may also lead to discrimination or derogation of non-group members (Brewer, 1999). The biological mechanisms that drive in-group favoritism and out-group prejudice are just beginning to be studied (Amodio et al., 2004; Knutson et al., 2007; Van Bavel et al., 2008). Some of this research has focused on the neuropeptide oxytocin (OT) because it facilitates attachment, social approach, and prosocial behaviors like trust and cooperation, as well as maternal defense (e.g., Zak et al., 2004; Kosfeld et al., 2005; Huffmeijer et al., 2013; Carter, 2014; Hostinar et al., 2014; Algoe et al., 2017).

### In-Group Bias

OT’s prosocial effects are likely to be depend to social context (e.g., Bartz et al., 2011; Shamay-Tsoory and Abu-Akel, 2016). OT has been shown to facilitate social recognition in human and non-human animals (Bielsky and Young, 2004) and to enhance the saliency of social cues (Pfundmair et al., 2017). Social salience, in turn, can increase prosocial behaviors that are facilitated through negative emotions like anger, leading to punishment of non-cooperative behaviors like free-riding (Aydogan et al., 2017). Social salience is the likely cause of the so-called “dark side” of OT, namely bias of one’s preferences toward in-group members (Shamay-Tsoory and Abu-Akel, 2016). Studies indicate that exogenous OT infusion promotes in-group (parochial) altruism (De Dreu et al., 2010; Ten Velden et al., 2017), ethnic in-group preference (De Dreu et al., 2011), protection of vulnerable in-group members (De Dreu et al., 2012), and the promotion of in-group norms (Daughters et al., 2017). Taken together, these studies show that OT promotes in-group preference rather than out-group derogation or hate (De Dreu, 2012; Shamay-Tsoory and Abu-Akel, 2016).

When drawing these conclusions, though, one needs to consider studies that question whether OT induces a bias against out-groups. For instance, OT given to Jewish Israelis increased empathy for pain experienced by Palestinian Arabs (Shamay-Tsoory et al., 2013). Notably, OT did not impact in-group empathy toward fellow Jewish Israelis. More generally, OT infusion appears to produce either prosocial or defensive behaviors depending on context, consistent with findings in animal studies (Bartz et al., 2011). Situational context is known to influence in-group/out-group behaviors (Mackie and Hamilton, 1993; Goette et al., 2012; LaBouff et al., 2012). Yet, studies using exogenous OT often pit an in-group against an out-group by asking people make decisions that explicitly benefit their group (De Dreu et al., 2010, 2011; De Dreu, 2012). These studies claim that OT preserves group membership by avoiding or possibly punishing out-groups (De Dreu, 2012). However, studies that do not stimulate group competition report that OT administration is associated with an increase in benefits for both in- and out-group members compared to placebo (Israel et al., 2012; Shamay-Tsoory et al., 2013; Huang et al., 2015). In a similar vein, a meta-analysis of OT infusion and trust found that OT increases in-group trust but does not reduce trust toward out-group members (Van Ijzendoorn and Bakermans-Kranenburg, 2012). The balance of evidence in the OT infusion and group literature indicates that exogenous OT increases the effect of primed group competition by intensifying a situational feature in the experiment. Absent a competition prime, OT is more likely to amplify what appears to be a moderate predilection for prosocial behaviors in humans.

Another factor that can affect how OT impacts group behavior is the use of groups formed in the laboratory, rather than studying existing groups. OT infusion appears to have a different effect when interacting with a known other compared to a stranger (Declerck et al., 2010, 2014). Using only randomly-formed groups to study biases may be another contextual feature that impacts extant OT findings. Further, a larger OT signal may be needed to motivate social interactions among strangers compared to known individuals (Wacker and Ludwig, 2012). Studies that examine endogenous OT release have only reported prosocial effects in psychologically healthy populations (Zak et al., 2005; Gonzaga et al., 2006; Morhenn et al., 2008; Barraza and Zak, 2009; Israel et al., 2009; Hurlemann et al., 2010; Crockford et al., 2014). In animals and humans, endogenous OT appears to be a response to a positive social stimulus and causes most people to reciprocate in a positive manner (reviewed in Zak, 2012).

### Endogenous Oxytocin

OT infusion studies seldom test if endogenous OT responds to the experimental stimulus. If we want to understand how the brain processes social information, best practice is to measure the response of endogenous OT and then confirm such a finding using exogenous OT. To date, studies examining the role of OT on in-group/out-group behavior have almost exclusively utilized exogenous OT infusion, with a few notable exceptions using less reliable endogenous OT analytes (urine, saliva). Urinary OT has been observed to increase before and during intergroup conflict in wild chimpanzees (Samuni et al., 2017). The increase in reactive OT was positively associated with greater group cohesion during intergroup conflict, but not the degree of out-group threat. A study examining Jewish-Israeli and Arab-Palestinian adolescents found a positive correlation between salivary OT concentrations and the extent of in-group bias (Levy et al., 2016). However, the positive correlation for OT and in-group bias only came from the Jewish-Israeli participants, and only for what the authors termed “neural in-group bias” defined as the amount of alpha modulation in the somatosensory cortex while empathizing with vicarious pain from in-group and out-group members. No results were reported on social behavior or self-reported bias toward the out-group and OT. Blood draws, if done rapidly because of OT’s approximately 3 min half-life, are the most effective way to capture the release of OT after a stimulus (Rydén and Sjöholm, 1969). While there are many ways to induce OT release, in every experiment with healthy adults, none generate this effect in every participant for a variety of reasons (Zak, 2012).

### Current Study

The studies of bias and OT do not provide a clear prediction on whether endogenous OT release will be associated with an in-group bias. Moreover, emerging research reveals a concern with the reliability and replicability OT infusion studies (Nave et al., 2015; Lane et al., 2016) and disagreements regarding how intranasal OT research should be interpreted (Churchland and Winkielman, 2012; Leng and Ludwig, 2016; Walum et al., 2016). These concerns show the need for a comprehensive approach to studying OT and social phenomena. We seek to do this in the present study by measuring the change in endogenous OT following interactions with group members, including both males and females in non-competitive tasks (i.e., allocations toward one group do not impact the other group), using a large sample size, and studying both previously-formed and randomly-formed groups.

## Materials and Methods

This study used group activities to stimulate endogenous OT release and relate the change in OT to in- and out-group bias. While basal plasma OT and central OT are unrelated, after stimulation, the change in OT in plasma and cerebral spinal fluid are positively correlated across several studies (Neumann et al., 2013; Valstad et al., 2017). Taking this into account, the analysis here only uses the percent change in OT in plasma to reflect the effects of central OT. In more than a decade of research measuring endogenous OT, we have found that social interactions that stimulate OT will only do so for a subset of participants (Zak, 2012). Our approach uses this finding to compare the behavior of participants who had an increase OT (OT+) to those for whom the interaction did not increase OT (OT−).

### Participants and Recruitment

Three hundred and ninety-nine participants were recruited from Claremont Graduate University, Westmont College, and local organizations within the Claremont and Santa Barbara communities. The sample size was based on size effects for OT release during monetary transfer tasks (Zak et al., 2005; Barraza and Zak, 2013). Two locations were used to increase the diversity of participants and group membership. Randomly formed groups were made up of 176 Claremont College students and 66 Westmont College students. These participants were randomly assigned to members of either “red” or “blue” groups (based on the minimal groups paradigm, Brewer, 1979; Lemyre and Smith, 1985; Ford and Stangor, 1992; Dunham et al., 2011). Previously formed groups included a group of local Claremont Colleges Reserve Officer Training Corps (ROTC) members (*N* = 30), a group of individuals from a student-led Claremont Colleges Christian organization (*N* = 27), a group of students from Westmont College (*N* = 56), and a group of Pentecostal church members recruited in Santa Barbara (*N* = 44). Sixty-four percent of the participants were Caucasian, 14% were Asian, 7% were Hispanic, 3% were African American, 3% described themselves as multi-ethnic, 7% described themselves as other, and 2% did not reveal their race. Participants were between the ages of 18 and 67 (with 82% between 18 and 22; *M* = 22.76, SD = 8.61). Fifty-three percent of participants were females. Recruitment for those in previously-formed groups (P) used target groups, and recruitment for randomly-assigned groups (R) focused on the broader population of students from the Claremont Colleges and Westmont College. This study was carried out in accordance with the recommendations of institutional review boards with written informed consent from all participants. All participants gave written informed consent in accordance with the Declaration of Helsinki. The protocol was approved by the institutional review boards at Claremont Graduate University and Westmont College.

### Procedures

After assignment to the red or blue groups, participants were given a random identification number on a paper badge in either blue or red ink to place on their chests for visibility. Color assignment was counterbalanced. After color assignment, participants completed trait surveys and provided a 12 ml blood sample obtained by a qualified phlebotomist to establish basal levels of OT.

After blood samples were obtained, groups were led into rooms segregated by color. Participants completed pre-task surveys, and a research assistant explained the group task. We did not want our findings to depend on a particular group task so we designed tasks that were ecologically valid for different groups. We expected that by making group membership salient, these tasks would stimulate OT release. R participants engaged in one of three group tasks. The first involved playing the game Scribblish; this game was chosen because it is noncompetitive, fun, and something people of all ages can do. Other R participants were asked to have a group conversation to get to know each other, or to sing folk songs with a leader who was not a participant. Tasks for those in P groups were also designed to reinforce group membership. These included marching for 15 min for the ROTC group, singing religious songs for 15 min with a song leader in the student Christian organization, and participating in a typical worship ceremony with a leader for 15 min for the Pentecostal church members. After the group task, participants completed post-task surveys and then provided a second 12 ml blood sample. Group tasks were staggered to reduce waiting time for the second blood draw (Zak et al., 2005). This allowed blood samples to be obtained from all participants within 5 min after the group task concluded. Next, participants were seated in a large computer lab with partitioned stations where they were instructed in and made monetary decisions. Once the decision tasks were finished, participants completed post-experiment surveys, were informed of their earnings in private, and were paid and released from the experiment.

### Materials

#### Pre-task Surveys

Participants were asked to complete a demographic survey that included questions on age, ethnicity and religious affiliation. Two surveys measured closeness to others and mood using the Inclusion of Other in Self (IOS; Aron et al., 1992) and the Positive Affect and Negative Affect Scale (PANAS; Watson et al., 1988). The PANAS asked participants to rate their current affective state on a scale from 1 to 5 (1 meaning they were currently feeling the emotion *very slightly or not at all*, and 5 meaning they were currently feeling the emotion *extremely*). The IOS asked participants about how close they felt to: (1) others in their group (red or blue); (2) something bigger than themselves; and (3) to their previously formed group when appropriate.

#### Post-task Surveys

The IOS, PANAS, Religious Commitment Inventory that refers to how much an individual is involved in religious activities (RCI, Worthington et al., 2003) and a survey we created on the context of one’s identification with their in-group (GROUPID) based on related research (Hogg et al., 1998) were given after the group task. The GROUPID survey asked participants to rate how much they favored their group on a scale from 1 to 5 (1 being *not very favorable* and 5 being *very favorable*) on seven dimensions (e.g., belonging, fit with one’s values) that were summed to create a GROUPID score.

#### Decision Making Tasks

To measure in-group and out-group bias, participants made monetary decisions involving people from both groups. In these tasks, participants made choices by computer in two rounds of the ultimatum game (UG), and dictator game (DG) as Decision-Maker 1 (DM1) and as Decision-Maker 2 (DM2). Participants were fully and identically instructed in each task, all decisions were double-blind, and there was no deception of any kind. Before each decision, participants were informed via software if their decision partner was a member of the red or blue group (i.e., was an in- or out-group member). All participants made choices in each task with both an in-group member and an out-group member and decisions were made in private in partitioned computer stations. Random assignment determined whether a participant was DM1 or DM2, and dyads were determined by random assignment. Pairings were not sustained across decision tasks to remove the effect of reputation and tasks were counterbalanced across sessions. Participants were informed that they would be paid 50 cents for every dollar they earned in the decision tasks described below.

In the UG, DM1 was endowed with $10 USD, while DM2 had nothing. The instructions stated that DM1 would be prompted to offer a split of the $10 to DM2. If DM2 accepted the split, the money would be paid to both DMs. If DM2 rejected the split, both DMs would receive $0. Both DMs were informed of this structure. After instruction and a chance to ask questions, DM1 was prompted by computer to enter the split proposal. At the same time, DM2 was prompted to report the minimum amount of money she/he was willing to accept from DM1. The software tallied the payoffs but these were not revealed to DMs so as to reduce possible experience effects. The UG requires the use of theory of mind (Camerer, 2003) and is used to measure selfishness and generosity (Zak et al., 2007).

**Figure 1:**
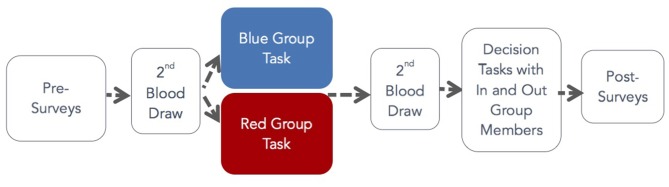
Experiment flow with randomization to group tasks.

In the DG, DM1 was endowed with $10 and DM2 had $0. The endowment amounts were common knowledge. After instruction, DM1 was prompted by computer choose how much, if any, of his or her $10 to transfer to the DM2 in the dyad. DM2 made no decision in this task. The DM1 transfer is thought to measure altruism (Smith, 1998). Figure 1 shows the flow of the experiment.

### Blood Handling

Blood was drawn from an antecubital vein using an EDTA (ethylenediaminetetraacetic acid) whole blood tube while maintaining a sterile field and using a Vacutainer^®^ (BD, Franklin Lakes, NJ, USA). Following the draw, blood tubes were rocked to facilitate mixing and prevent coagulation and were immediately placed on ice. Within 15 min, tubes were centrifuged at 1500 rpm for 12 min at 4°C following our published protocol (Zak et al., 2005). Plasma was removed from the tubes with disposable pipettes and placed into 2 ml microtubes with screw caps. These tubes were immediately placed on dry ice and stored at −80°C until assays were performed.

OT was assayed from plasma using an RIA (radioimmunoassay) kit produced by Bachem, Incorporation (Torrance, CA, USA) in duplicate including an extraction step. The RIA has been shown to be more reliable at detecting OT than an ELISA (enzyme-linked immunosorbent assay), with extraction as a necessary step in the process (McCullough et al., 2013; Christensen et al., 2014). The inter- and intra-assay coefficients of variation for OT were 4.58% and 4.01%, and detection levels were 0.5 pg/ml. OT was assayed at the Reproductive Endocrine Research Laboratory at the University of Southern California (Los Angeles, CA, USA). Ten outliers (>3SD over mean) in basal OT or stimulated OT were removed from the sample and on inspection the percent change in OT was normally distributed.

### Statistical Analysis

Independent *t*-test were utilized to examine the extent of bias shown toward the in-group and out-group for decision tasks and how OT release affected this decision. We examined the context of decisions using independent *t*-test to examine differences between those from previously formed groups vs. randomly formed groups. We analyzed the overall impact of group type (P or R), OT (OT+, OT−), and group identification (GROUPID) using a linear regression model. This model was also used to determine the extent that personality traits affected bias.

## Results

Of the 399 participants, 11 did not have complete blood data, 17 did not complete the monetary decisions tasks, and 53 were missing survey data for the GROUPID questionnaire. Participants with missing data were used in all analyses except for in cases where their data was missing. Table 1 has descriptive statistics for the sample.

**Table 1:** Descriptive statistics for Oxytocin + (OT+) and OT− groups and for previously (P) and randomly (R) formed groups.

Variable	OT+	OT−	P	R
*N*	205	180	157	239
Age	22.41 (8.73)	22.95 (8.49)	23.70 (10.18)	22.13 (7.35)
Gender	50% female	58% female	55% female	53% female
In-group UG DM1	5.24 (1.82)	5.29 (1.94)	5.75 (2.19)	4.93 (1.52)
Out-group UG DM1	5.26 (1.90)	4.98 (1.75)	5.54 (2.08)	4.83 (1.58)
In-group UG DM2	2.38 (1.76)	2.34 (1.85)	2.19 (18.6)	2.50 (1.75)
Out-group UG DM2	2.36 (1.70)	2.45 (1.98)	2.31 (1.92)	2.49 (1.77)
In-group DG DM1	4.34 (2.75)	4.21 (2.65)	4.97 (2.85)	3.79 (2.53)
Out-group UG DM1	3.88 (2.84)	3.72 (2.74)	4.40 (2.97)	3.40 (2.61)

*Values in parentheses are standard deviations*.

### Overall Bias

When considering the entire sample, more money was transferred to in-group members compared to the out-group participants for all DM1 decisions (UG DM1: in-group *M* = 5.26, SD = 1.87, out-group *M* = 5.12, SD = 1.84, paired *t*_(381)_ = 2.26, *p* = 0.025, 95% CI [0.08, 0.25]; DG: in-group *M* = 4.26, SD = 2.72, out-group *M* = 3.82, SD = 2.80, paired *t*_(381)_ = 5.51, *p* < 0.001, 95% CI [0.28, 0.60]).

### Bias by Group Type

As we expected, P participants gave more to their in-group compared to their out-group in all decisions except as DM2 in the UG. Those in the R group gave more money to their in-group in the DG, but not as DM1 and DM2 in the UG (*p* > 0.05). These biases are partially attributable to a stronger contextual identification (GROUPID) for P vs. R participants (P: 3.84, SD = 0.83 R: 3.44, SD = 0.69, *t*_(194.13)_ = −4.48, *p* < 0.001, 95% CI [−0.58, 0.23]). GROUPID was positively correlated with in-group bias by DM1s in both decision tasks (UG: *r* = 0.12, *p* = 0.034; DG: *r* = 0.12, *p* = 0.035). Bias was unrelated to group closeness (IOS) or changes in mood (PANAS).

### Oxytocin Stimulation

**Figure 2:**
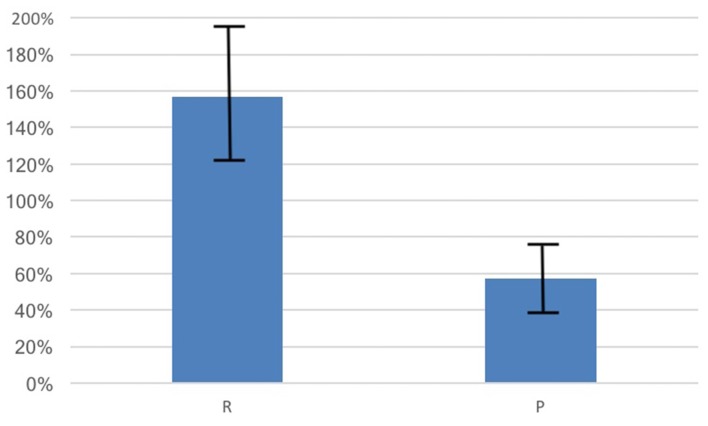
OT increased from baseline due to social interactions by 157% for those in therandomly-formed group (R) while participants in the previously-formedgroup (P) had an OT increase of 57%. The change in OT for the R group is significantly larger than for the *P* group (*p* = 0.012). Bars shown are standard errors.

Average basal OT was in the expected range (*M* = 5.97 pg/ml, SD = 12.75) and the average percentage change in OT was positive (*M* = 116.09%, SD = 452.40%, *t*_(387)_ = 5.06, *p* = 0.004, 95% CI [70.93, 161.25]). Consistent with our hypothesis, the percentage change in OT for those in randomly-formed groups (*M* = 156.59%, SD = 567.80%, *N* = 231) showed a significantly larger increase than for those in the previously formed groups (*M* = 56.50%, SD = 162.49%, *N* = 157, *t*_(282.72)_ = 2.53, *p* = 0.012, *d* = 0.22, 95% CI [22.24, 177.93], see Figure 2).

Fifty-two percent (*N* = 205) of participants showed an increase in OT (OT+) following the group task. Among these individuals, the average increase was 251.57%, which was significantly different from zero (*t*_(207)_ = 6.20, *p* < 0.001, 95% CI [171.62, 331.53]). As above, OT+ participants in randomly-formed groups had a larger increase in OT than those in previously formed groups (R: *M* = 526.65%, SD = 1089.51; P: *M* = 273.28%, 699.07; *t*_(203.62)_, 2.04, *p* = 0.043, 95% CI [8.47, 498.27]).

### Oxytocin and Bias

**Figure 3:**
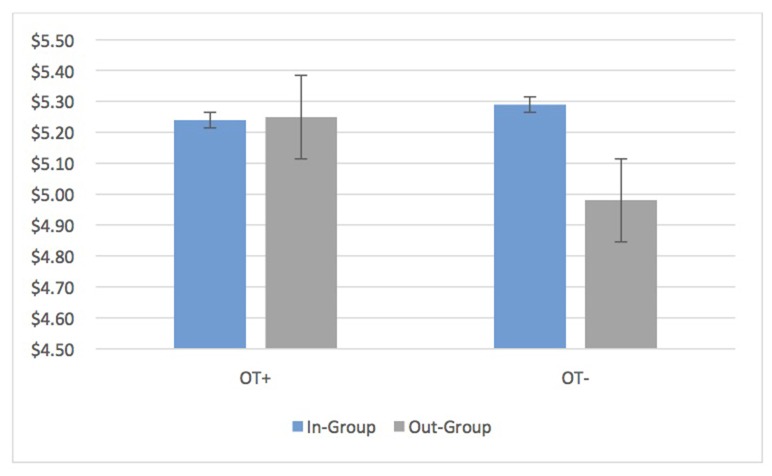
OT+ participants have identical average transfers to in-group and out-group members in the ultimatum game (UG) while OT− participants show an average bias of 6.2% ($0.31) towards in-group members. Bars are standard errors.

Average transfers by OT+ as DM1s in the UG showed no bias at all (OT+ In: 5.24, SD = 1.82 Out: 5.25, SD = 1.90, *t*_(202)_ = −0.20, *p* = 0.84, 95% CI [−0.16, 0.13]). OT− participants continued to have in-group bias in the UG and DG (DM1 UG In: 5.29, SD = 1.94 Out: 4.98, SD = 1.77; *t*_(173)_ = 3.09 *p* = 0.002, 95% CI [0.11, 0.50]; DG DM1 In: 4.21, SD = 2.65, Out: 3.78, SD = 2.73, *t*_(171)_ = 3.50, *p* = 0.001, 95% CI [0.19, 0.67]; Figure 3). Put differently, the relative in-group bias in the UG (In-group transfer—Out-group transfer) disappeared for OT+ while it was sustained for OT− (OT+: *M* = −0.015, SD = 1.30, OT− *M* = 0.31, SD = 1.61; *t*_(331.68)_ = 2.59, *p* = 0.01, 95% CI [0.08, 0.56]). Nevertheless, an in-group bias continued to appear for OT+ for unilateral transfers in the DG (In: $4.34, SD = 2.75, Out: $3.88, SD = 2.84; *p* < 0.001, 95% CI [0.25, 0.68]). When it came to reciprocation (UG DM2), there was no bias in the minimum acceptable offer for OT+ and OT− (OT+: *M* = 0.015, SD = 0.952; OT−: *M* = −0.139, SD = 0.750; *t*_(374)_ = −1.72, *p* = 0.087, 95% CI [−0.33, 0.02]).

To isolate the effects of OT, a linear regression model using group type (previously-formed or randomly-formed) and binary indicator for OT+ or OT− to explain DM1 in-group bias (in-group transfer minus out-group transfer) was estimated for both decisions tasks. Age and gender were included as covariates. OT+ was negatively related to in-group bias across both tasks (*R*^2^ = 0.03, *F*_(4,368)_ = 3.06, *p* = 0.017; *b* = −0.293, *β* = −0.12, *t*_(368)_ = −2.40, *p* = 0.017). Age and gender were not significant and the OT+ indicator continues to be significant without their inclusion. Group type was also insignificant (*p* = 0.31).

When GROUPID was added to the regression model, it significantly increased in-group bias (*R*^2^ = 0.04, *F*_(5,324)_ = 2.32, *p* = 0.043; *b* = 0.174, *β* = 0.12, *t*_(324)_ = 2.18, *p* = 0.03) even though GROUPID and the OT indicator are not correlated (*r* = −0.069, *p* = 0.209). We also tested the role of religion on bias since some of the previously-formed groups had religious members. We created the indicator variable REL that took the value of 1 if the participant’s score on the RCI exceeded the median. The group-type indicator was dropped from model because of its high correlation with REL (*r* = 0.796, *p* < 0.001) and the model was re-estimated. REL was insignificant (*β* = 0.07, *p* = 0.163) while the OT indicator remained significant (*β* = −0.13, *p* = 0.015). Average values for GROUPID, REL, closeness to those in one’s group (IOS) or mood (PANAS) at baseline, after the group task, or pre-to-post change showed no differences when comparing OT+ participants to OT− ones. We examined the degree of group identification required to overwhelm the impact of a positive change in OT producing a bias towards one’s in-group. Using the regression of in-group bias on the OT+ indicator and GROUPID, in-group bias occurs when GROUPID is one standard deviation above the mean, or 87% of its maximum value.

We also tested if personality traits might vary across the OT+ and OT− groups and might affect our findings. We found that, on average, those in the OT+ group were less agreeable (OT+: *M* = 4.01, SD = 0.60, OT−: *M* = 4.16, SD = 0.63; *t*_(375)_ = 2.29, *p* = 0.022), were less neurotic (OT+: 2.41, OT−: 2.67; *t*_(375)_ = 3.16, *p* = 0.002), reported less empathic concern (OT+: *M* = 3.85, SD = 0.64, OT−: *M* = 4.04, SD = 0.60; *t*_(374)_ = 2.87, *p* = 0.004), and more personal distress (OT+: *M* = 2.46, SD = 0.70, OT−: *M* = 2.64, SD = 0.70; *t*_(376)_ = 2.40, *p* = 0.017). When traits were added to the linear regression model, none of the trait variables were significant (*ps* > 0.15) and OT+ and GROUPID continued to be significant and had similar beta coefficients to the regression without the trait measures.

## Conclusions and Discussion

The present study investigated the relationship between in-group bias and endogenous OT in a non-competitive environment using previously-established groups and randomly-formed groups. Research using exogenous OT administration has suggested that OT increases in-group bias in competitive contexts (De Dreu et al., 2011; De Dreu, 2012) but may decrease bias when competition is not explicit (Israel et al., 2012; Shamay-Tsoory et al., 2013; Huang et al., 2015). Whether the endogenous release of OT affects group bias was an open question, with only a few studies on the topic (Levy et al., 2016; Samuni et al., 2017). We found that half of the 399 participants had a positive increase in endogenous OT after a group activity and OT+ participants showed no bias as DM1 or DM2 in the UG, though they did show bias in the DG. OT− participants were biased as DM1 in both decision tasks. While the UG is a bilateral social interaction in which both parties make choices, in the DG only one person makes a decision. Indeed, transfers in the DG do not appear to be affected by OT infusion (Zak et al., 2007; Barraza et al., 2011) perhaps because the other person’s needs do not need to be considered in relation to the self.

Our results show that the effect of OT on bias is context-dependent (Bartz et al., 2011). Endogenous OT, even when group membership was made salient across the various types of groups we studied, seems to generally reduce group differences, although not fully eliminate bias when group identification was high (87% of maximum value or higher). As argued by others (Shamay-Tsoory and Abu-Akel, 2016), OT may benefit out-group members when there is a strong social cue, or when group status is highly-charged as in the Israeli-Palestinian conflict (Shamay-Tsoory et al., 2013). Consistent with a large literature on the prosocial effects of OT, we showed that an increase in endogenous OT eliminated bias in the UG, a task that motivates others to think about the other player whether in-group or out-group. This was true for both previously-formed groups and randomly-formed groups. Behaviorally, those in P groups had a larger in-group bias than R participants because they identified more strongly with people they already knew or a group to which they belonged. Yet, when OT increased, the bias from being a member of a previously-formed group largely disappeared even though the strength of group identification diminished out-group transfers. This result held even when accounting for personality traits. The motivation for perspective-taking is relatively absent in the DG and bias in the DG was unrelated to OT reactivity. Future studies should examine whether similar social cuing impacts group biases. A related study has shown that there are no in-group/out-group saliency differences during the early stages of information processing (Pfundmair et al., 2017).

There are two caveats when considering research utilizing peripheral plasma measures of OT. First, much like methods in OT administration, OT plasma assays methods have come under criticism. Commercially available immunoassays have been questioned on their validity due to high variability (e.g., McCullough et al., 2013; Christensen et al., 2014; Rutigliano et al., 2016). These same authors report that using the methods utilized in this study (radioimmunoassay along with an extraction step) reduces this high variability (e.g., Szeto et al., 2011; Christensen et al., 2014). A second concern in measuring peripheral plasma OT is in attributing the levels to central OT (McCullough et al., 2013). The most recent meta-analysis has found that peripheral and central OT concentrations are positively correlated, but only after an environmental stimulus and not under basal conditions (Valstad et al., 2017). Future research is needed to identify the types of environmental stimuli that lead to a connection between peripheral and central OT concentrations.

The present study also advances knowledge about group bias by using a large and diverse participant population, tested in two locations, and using ecologically valid group tasks to make group membership salient. This approach increases the likelihood that our results will replicate. This is especially important given the small effect sizes noted in exogenous OT infusion studies (Walum et al., 2016). Additional research should also test participants from non-Western societies to see how OT modulates group biases because of differences found in the behavioral expression of the OT receptor system across ethnicities (Kim et al., 2010).

## Author Contributions

PJZ and JS: funded, designed, analyzed and wrote the study. ETT and LEB: executed study, analyzed data and wrote the findings. JAB: executed, designed, analyzed and wrote the study.

## Conflict of Interest Statement

The authors declare that the research was conducted in the absence of any commercial or financial relationships that could be construed as a potential conflict of interest.
